# Chimeric tricomposite flap from the second dorsal metacarpal artery

**DOI:** 10.1016/j.jpra.2025.04.004

**Published:** 2025-04-13

**Authors:** Célina Cottier, Sébastien Durand

**Affiliations:** Department of Hand Surgery, Lausanne University Hospital and University of Lausanne, Avenue Pierre-Decker 5, 1011 Lausanne, Switzerland

**Keywords:** Chimeric flap, Dorsal metacarpal artery, Extensor indicis proprius, Vascularized tendon, Vascularized bone graft

## Abstract

Reconstructing multi-tissue defects in the finger remains a significant challenge in hand surgery. We present the case of a 37-year-old man with segmental loss of bone, skin and extensor apparatus on the dorsal aspect of the index finger. A single stage reconstruction was successfully performed using a pedicled chimeric flap based on the second dorsal metacarpal artery combining skin paddle, second metacarpal base bone and the extensor indicis proprius. At 12 weeks, proximal interphalangeal joint fusion was achieved and the skin flap healed uneventfully. The patient regained full extension and 20° of active flexion at the distal interphalangeal joint. To our knowledge, this specific chimeric flap has not been previously described in the literature. This technique provides a versatile, single-stage solution for complex dorsal finger reconstruction, minimizing morbidity and optimizing functional outcomes.

## Introduction

The second dorsal metacarpal artery (2DMA) is consistent vascular structure with origins from the dorsal intercarpal arch (23 %), a perforating branch of the deep palmar artery (13 %) or a mixed origin (63 %).^1^ It runs along the second web space, adjacent to the second metacarpal on its ulnar border and beneath the extensor indicis tendons. Distal anastomoses between the dorsal metacarpal artery and the palmar network are mostly located at the level of the metacarpal heads.^1^ The 2DMA has an average of six branches, including one constant distal cutaneous branch located an average of 1.2 cm^2^ proximally to the metacarpophalangeal (MCP) joint when the 2DMA reaches the distal margin of the junctura tendinae. Another branch, usually 3–4 cm upstream from the MCP joint, supplies the extensor indicis proprius (EIP)^3^ and an average of three branches supply the second metacarpal bone.^1^

Complex finger traumas involving multi-tissue loss pose a significant challenge in reconstructive hand surgery. Previous studies have described a distal pedicled composite flap from the 2DMA, including a second metacarpal base bone graft and skin paddle for index finger reconstruction.^3^^,^^4^ However, these injuries are often associated with tendon loss. Vascularized tendon transfer is particularly advantageous for repairing traumatic tendon defects or failed primary repairs. It preserves the peritendinous structures and blood supply, potentially reducing adhesion, minimizing apoptosis, and promoting accelarated healing.^5^^,^^6^

We report the case of a chimeric tricomposite flap from the 2DMA incorporating skin, bone and vascularized tendon for complex dorsal finger reconstruction. To our knowledge, this is the first report of this specific technique.

## Case report

A 37-year-old male carpenter sustained severe hand trauma from a spindle moulder accident, resulting in distal thumb amputation and extensive damage to the index and middle fingers. The index finger exhibited skin loss, extensor apparatus disruption, and bone loss at the proximal interphalangeal (PIP) joint, while the middle finger had an open intra-articular PIP fracture.

**Figure 1:**
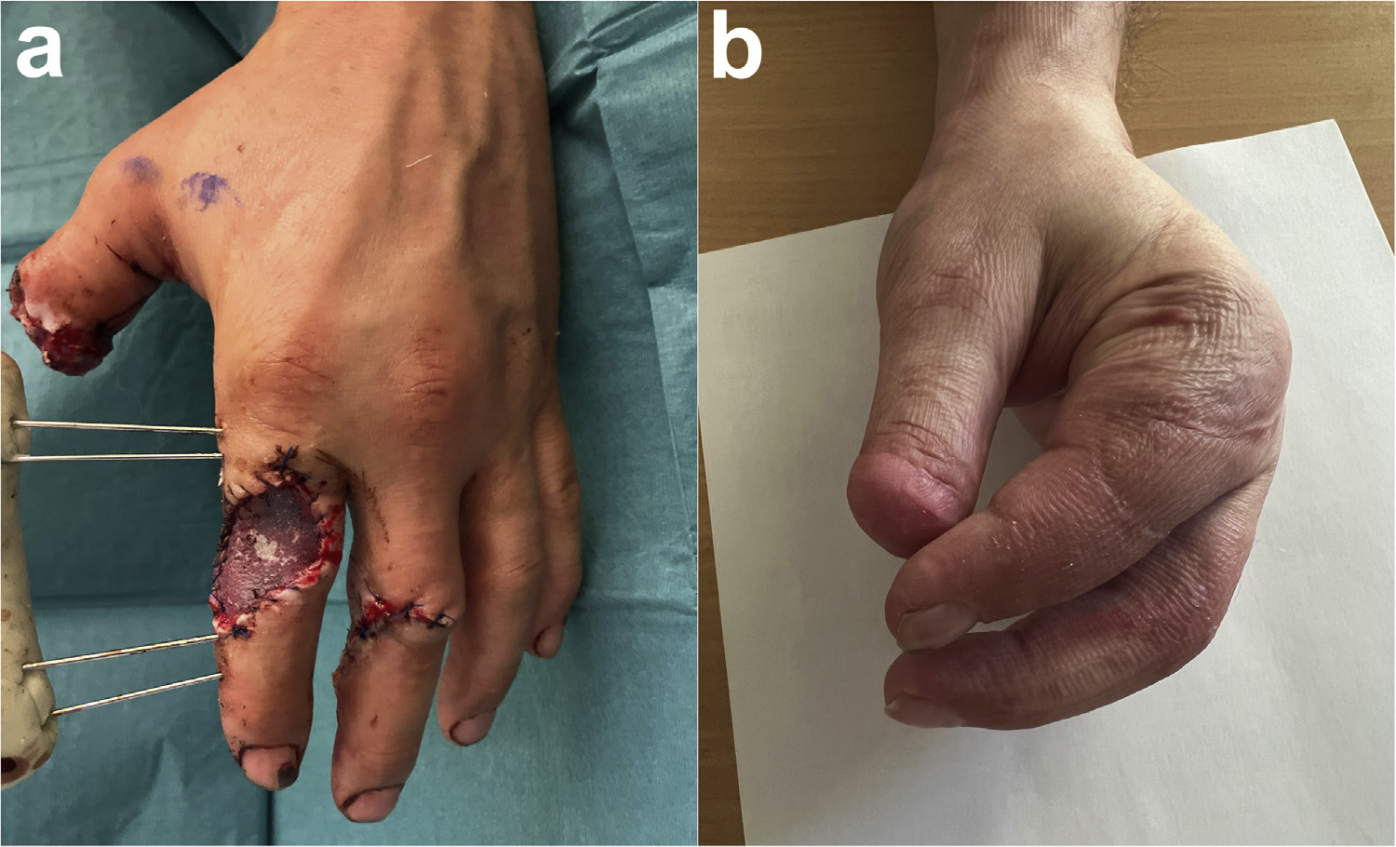
Initial debridement and temporary wound coverage with EpiGARD^Ⓡ^, external fixation stabilized the index finger (a). Clinical results 3 months after surgery (b).

**Figure 2:**
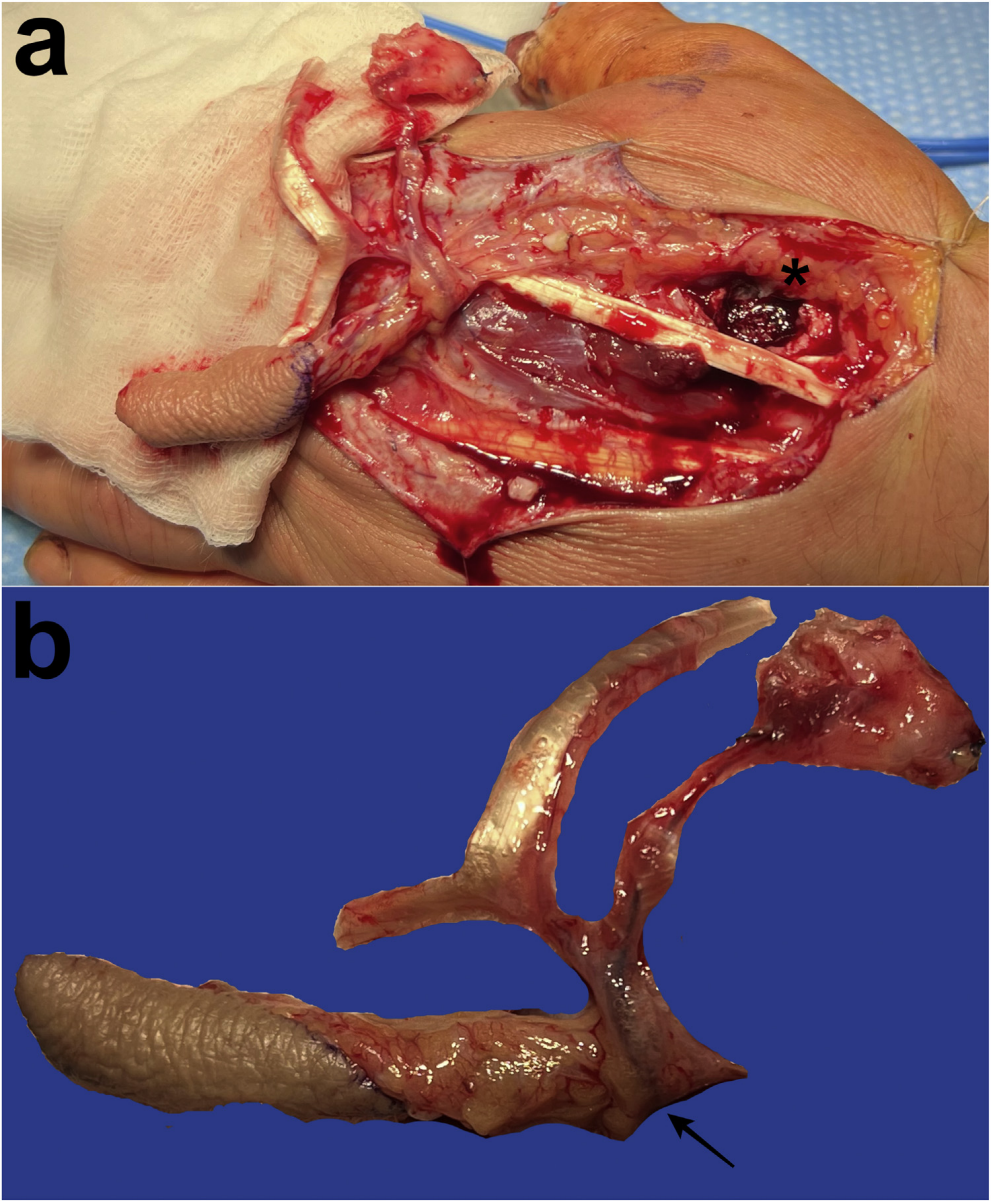
Intraoperative photographs showing the 2DMA (arrow) chimeric tricomposite flap including a vascularized skin paddle, bone from the second metacarpal base (asterisk) and EIP tendon (a and b).

After initial debridement and temporary wound coverage with EpiGARD^Ⓡ^ (Biovision GmbH, Ilmenau, Germany), external fixation was used to stabilize the index finger (Figure 1). Two days later, a chimeric tricomposite flap (Figure 2) was performed under tourniquet control to prevent exsanguination while ensuring clear visualization of the 2DMA and its vascular branches.

**Figure 3:**
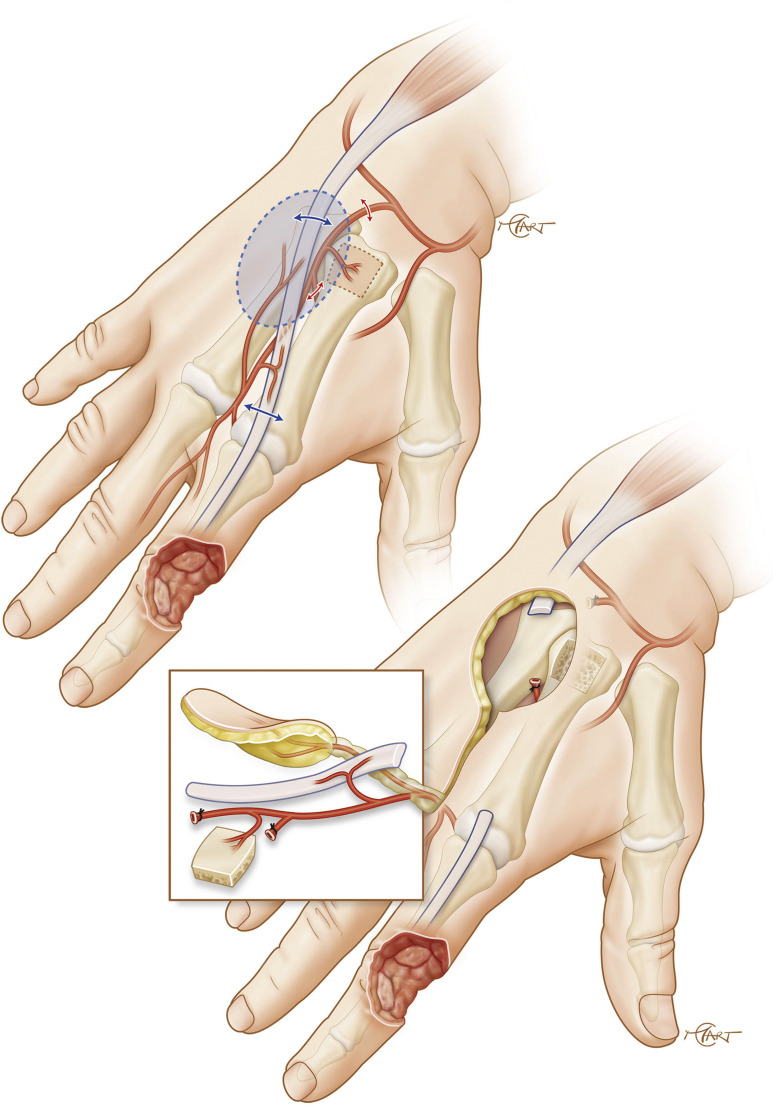
Arterial anatomy, ligation of several arterial branches is necessary to allow the chimeric flap to reach the PIP joint of the index.

Surgery was carried out by the senior author (S.D.). A skin paddle was outlined just distal to the bases of the adjacent metacarpal bones (Figure 3). Skin and subcutaneous tissue were incised and elevated from the second intermetacarpal space. Distal to the skin paddle, subcutaneous tissue covering the interosseous muscle was raised in the pedicle, excluding the fascia. The pivot point was located at the distal margin of the junctura tendinae, where the cutaneous branch reaches the 2DMA.

Deeper, the dorsal carpal arch, the 2DMA and the periosteal branches to the second metacarpal base were identified. The second carpometacarpal joint was delineated using fluoroscopy. A 1 × 1 cm vascularized bone graft was harvested using a 1-mm K-wire and osteotome ensuring that the cancellous bone remained attached to the cortex. The 2DMA was ligated proximally to the periosteal branches and its dissection was carried out distally up to the perforating branch from the palmar network at the level of the metacarpal neck. Precautions were taken to preserve the artery supplying the EIP. Dissection including the EIP, its gliding tissue and vascularization, was performed by carefully separating the EIP and the extensor digitorum communis (EDC) tendon intended for the index finger.^5^ The tendon was cut proximally and distally and rotated 180° with the skin paddle and the bone to fill the defect.

Bone grafting and Aptus^Ⓡ^ plating (Medartis AG, Switzerland) secured the PIP arthrodesis. The EIP was sutured distally to the terminal extensor mechanism and proximally to the intrinsic and extrinsic extensor remnants. The skin paddle was then sutured, and Splinting was applied continuously for three weeks postoperatively.

No donor or recipient site complications were observed. Bone union was achieved within 12 weeks and the skin flap healed uneventfully. At three months, the patient had full extension and 90° active flexion of the MCP joint, as well as full extension and 20° active flexion at the distal interphalangeal (DIP) joint. Patient was painless and satisfied with functional mobility despite thumb shortening.

## Discussion

Since an island pedicle cutaneous flap from the dorsum of the index finger was first described in 1979^7^ further experience has been documented using the 2DMA as a pedicle of the cutaneous flap.^8^ The advantages of 2DMA cutaneous flap include reliable vascularity with a large arc of rotation, allowing versatile reconstruction. Its elastic and thin skin makes it adaptable for finger resurfacing, while its minimal donor-site morbidity and good primary closure potential further enhance its clinical utility.

Isolated vascularized second metacarpal base bone graft was first described in 2000^9^ and numerous reports have demonstrated its reliability and feasibility, with a consistent 100 % union rate in different clinical settings and a relatively low morbidity.^3^^,^^9^ Although the flap size is limited, its wide arc of rotation provides great versatility. While previous publications have described its use in scaphoid nonunion and Kienböck’s disease, more recent studies have focused primarily on finger reconstruction.^3^

While vascularized bone grafts are well-established, vascularized tendon remains underutilized. The transfer of vascularized flexor digitorum superficialis (FDS) tendon, along with its gliding tissues has shown promise but requires sacrificing the ulnar artery.^5^^,^^6^ Vascularized EIP transfer offers an excellent alternative, as EIP is the preferred non-vascularized tendon graft when palmaris longus is absent.^10^

Previous cases^3^^,^^4^ have described the use of 2DMA chimeric flap, which includes both the second metacarpal base bone and skin. In one case, the cutaneous nerve in the skin flap was used to repair the digital nerve.^4^ These cases resulted in satisfactory outcomes without complications.

The 2DMA chimeric tricomposite flap represents a versatile, single-stage solution for reconstructing complex dorsal finger defect and follows the principle of “replace like with like”, embodying a biomimetic approach to tissue repair. The technique is relatively straightforward, with no need for microsurgical sutures. This first reported technique integrating a vascularized skin paddle, metacarpal bone and tendon minimizes morbidity and optimizes functional outcomes. Further research is warranted to explore its broader applications in hand surgery.

## CRediT authorship contribution statement

**Célina Cottier:** Writing – review & editing. **Sébastien Durand:** Conceptualization, Methodology, Writing – review & editing.

## Conflict of interest

None of the authors has conflict interest for the work done in this manuscript or for financing the study.
